# Uterine mesh compression suturing for refractory postpartum hemorrhage: a retrospective study of 45 cases

**DOI:** 10.3389/fmed.2025.1675709

**Published:** 2025-12-12

**Authors:** Dan Li, Chendi Wang, Li Li, Xu Yang

**Affiliations:** 1 Department of Obstetrics, The Fifth People's Hospital of Chengdu, Chengdu, China; 2 Department of Obstetrics, Wenjiang Maternal and Child Health Hospital, Chengdu, China

**Keywords:** indications, obstetric emergency, refractory postpartum hemorrhage, surgical efficacy, uterine mesh compression suturing

## Abstract

**Objective:**

To evaluate the clinical application and efficacy of a novel suturing technique—uterine mesh compression suturing—in the treatment of refractory postpartum hemorrhage.

**Methods:**

A retrospective analysis was performed on the clinical data of 45 patients with refractory postpartum hemorrhage who underwent uterine mesh compression suturing. The surgical efficacy and safety were systematically evaluated.

**Results:**

Active uterine bleeding was controlled in all patients within 10 min postoperatively. The cumulative vaginal bleeding volume at 2 h post-surgery was less than 20 mL, indicating effective hemostasis. MRI on postoperative day 4 revealed scattered punctate signals in the uterine wall, with no intrauterine fluid accumulation. At 42 days post-surgery, MRI showed a uniform signal in the myometrium and a clear endometrial line. Ultrasound at 42 days post-surgery demonstrated normal uterine size, uniform myometrial echogenicity, and good healing. Hysteroscopy at 6 months post-surgery revealed no intrauterine adhesions and clear fallopian tube ostia. All 45 patients resumed normal menstrual cycles 1–2 months after cessation of breastfeeding. Ten patients became pregnant again postoperatively: four underwent repeat cesarean deliveries, and six had artificial abortions.

**Conclusion:**

Uterine mesh compression suturing is an effective method for controlling refractory postpartum hemorrhage. This technique can be used as an intervention for patients with refractory postpartum hemorrhage unresponsive to traditional hemostatic methods, or those with blood loss ≥1,500 mL during cesarean section. It holds promise for broader clinical application.

## 1 Introduction

Postpartum hemorrhage (PPH) remains a leading cause of morbidity and mortality among pregnant women worldwide, accounting for 27% of maternal deaths (1). In 2020, the incidence of severe postpartum hemorrhage in China was still 0.96% (2). In developed countries, the incidence of postpartum hemorrhage is rising. Over the past decade, postpartum hemorrhage has been the leading cause of maternal mortality in Australia, with a maternal mortality rate of 4 per million (3). The four major causes of postpartum hemorrhage are uterine atony, birth canal injuries, placental factors, and coagulation disorders (4). Refractory postpartum hemorrhage refers to severe postpartum bleeding that cannot be controlled by conservative measures such as uterotonic drugs, continuous uterine massage, or uterine packing. It requires surgical intervention, interventional treatment, or even hysterectomy. For refractory postpartum hemorrhage caused by uterine atony, placental factors, and coagulation dysfunction, where uterine massage and uterotonics are ineffective and hysterectomy may be required, traditional surgeries often involve B-Lynch suturing (5). However, B-Lynch suturing may fail, and there is a risk of uterine infection and tissue necrosis postoperatively, necessitating strict adherence to surgical indications. Our hospital has introduced an innovative uterine suturing technique, uterine mesh compression suturing, primarily used for refractory postpartum hemorrhage. Between January 2014 and December 2023, 45 patients with refractory postpartum hemorrhage at our hospital underwent this procedure, with a 100% success rate in preserving the uterus. This article reviews the application of uterine mesh compression suturing in 45 cases over the past 10 years at our hospital, exploring its clinical value and providing a potential treatment option for patients with refractory postpartum hemorrhage.

### 1.1 General information

Inclusion criteria for patients who underwent mesh compression suturing: ① Postoperative bleeding ≥1,500 mL within 24 h after cesarean section; ② Postpartum hemorrhage caused by uterine atony; ③ Failure of hemostasis by conservative measures such as uterine massage, uterotonic drugs, or uterine packing.

Exclusion criteria: ① Exclusion of birth canal injury; ② Coagulation disorders as the cause of bleeding; ③ Complete placental implantation.

All patients obtained approval from the hospital’s ethics committee, and informed consent was obtained from the patients’ families prior to surgery, The 45 patients who underwent mesh compression suturing were divided into three groups based on the amount of bleeding: Group I: Patients with bleeding between 1,500 mL and 2,500 mL (16 cases). Group II: Patients with bleeding between 2,500 mL and 3,500 mL (15 cases). Group III: Patients with bleeding ≥3,500 mL (14 cases). Details of the grouping are shown in Table 1.

**Table 1:** Clinical data of 45 patients undergoing uterine mesh compression suturing.

Clusters (Bleeding)	Group I (16 cases) bleeding ≥ 3,500 mL	Group II (15 cases) 3,500 mL > bleeding ≥ 2,500 mL	Group III (14 cases) 2,500 mL > bleeding ≥ 1,500 mL
Average age (years)	28.5 ± 5.8	28.3 ± 6.6	28.0 ± 6.2
Average age at conception (times)	2.3 ± 1.5	2.1 ± 1.7	2.2 ± 1.3
Average number of births (times)	1.5 ± 0.6	1.1 ± 1.3	1.3 ± 1.2
Average week of pregnancy (weeks)	38.5 ± 3.6	38.3 ± 3.2	38.0 ± 3.1
Preoperative diagnosis			
Scarred uterus	6	5	3
Placenta praevia	8	7	6
Eclampsia	2	1	2
GDM	3	2	2
ICP	1	2	1
Abruption of the placenta	1	1	0
Giant baby	2	0	2
Abnormal fetal position	2	2	1
Twin pregnancy	2	0	1
Induce labor from stillbirth	1	0	0
Intraoperative diagnosis			
Placental implantation	8	7	6
Lack of contractions	12	10	9
Scarred pregnancy		1	
Uterine rupture	1		
Mode of delivery			
Cesarean section delivery	13	12	14
Vaginal delivery	3	2	0
Cesarean section and embryo removal		1	
Average bleeding	4,200 ± 364.4	2,960 ± 231.55	1781 ± 160.12
Other interventions	Contraction drugs, uterine massage, uterine artery ligation, local sutures, uterine tamponade, B-Lynch suture	Contraction drugs, uterine massage, local sutures, uterine tamponade	Contractions drugs, uterine massage, local sutures
Transfer to ICU	16	10	4

### 1.2 Surgical method

Instruments: No. 0 absorbable suture, 1/2 curvature, 10 × 24 round needle.

#### 1.2.1 Pre-suturing preparation

Before suturing, cleanse the uterine cavity and abdominal area of blood, and move the uterus outside the abdominal wall incision. Perform uterine massage, inject uterotonic drugs into the uterine body, and inspect the uterine body and lower segment. If placental implantation is present, perform placental separation, followed by “8”-shaped or interrupted sutures for hemostasis.

#### 1.2.2 Uterine mesh compression suturing method

① First suture: Insert the needle 3 cm from the lower edge of the uterine incision and 3 cm from the left side of the uterine body. Pass the needle through the uterine cavity, exiting 3 cm from the upper edge of the incision and 4 cm from the left side of the uterine body. Insert the needle at 3 cm intervals along the longitudinal direction of the uterine body, continuing the suture longitudinally through the uterine wall for 3 cm before exiting the needle. When the suture reaches 4 cm from the uterine fundus and 4 cm from the side margin, exit the needle. The suture will pass over the uterine serosal surface, around the uterine fundus, and towards the posterior wall (avoiding injury to the uterine cornua and fallopian tube openings). The suture will then turn horizontally to the right posterior wall. The remaining suturing will mirror the left side.

② Second suture: The insertion point is 3 cm above the first suture on the left side and 3 cm horizontally from the first suture. The direction of the suture from the anterior to posterior wall will follow the same path as the first suture, with the exit point corresponding to the left side insertion point.

③ Third suture (if needed): If the distance between the second suture and the uterine body exceeds 9 cm, a third suture is placed. The insertion and exit points for this suture will be 3 cm above the line connecting the entry and exit points of the second suture and 3 cm horizontally from the second suture. The direction of the suture from the anterior to posterior wall will follow the same direction as the first two sutures.

#### 1.2.3 Suture knotting

**Figure 1:**
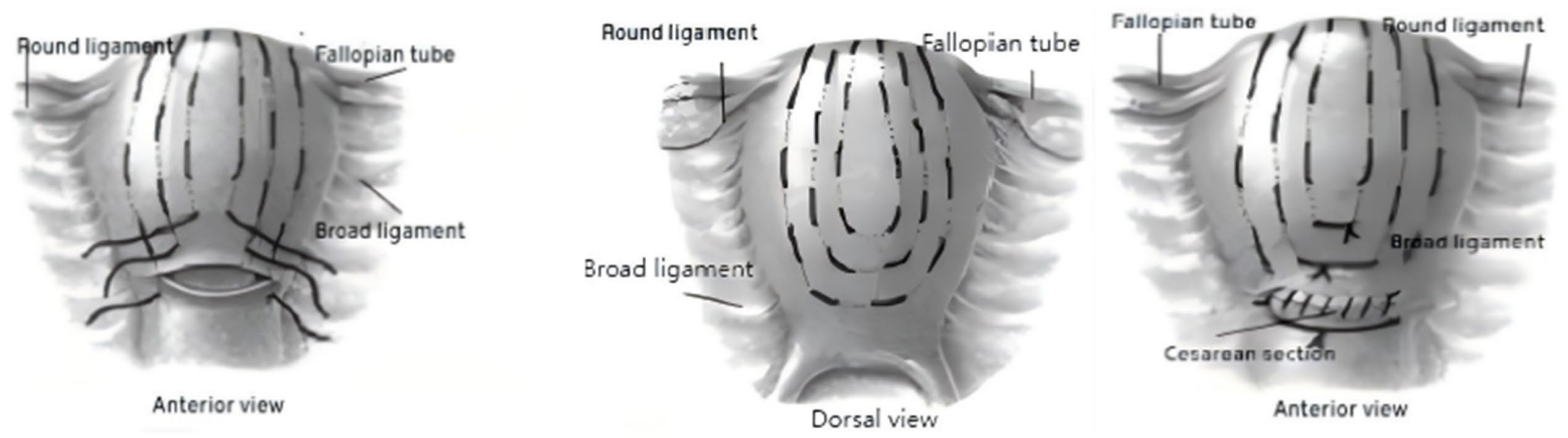
Schematic diagram of uterine mesh compression suturing.

**Figure 2:**
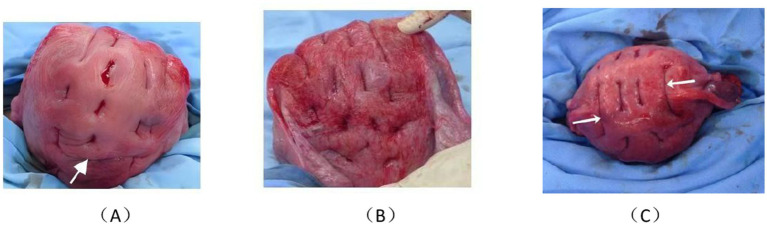
Postoperative photograph of the uterine mesh compression suturing. **(A)** Anterior view, with arrows indicating knotting of the second suture. **(B)** Dorsal view. **(C)** Fundal view, with left and right arrows showing the first suture crossing the fundus twice at the plasma level, maintaining a distance from the uterine horn to protect the tubal opening.

After tightening and knotting the third and second sutures, close the uterine incision with the first suture. The final knot for the first suture is tied at the lower segment of the uterine incision (see Figures 1, 2).

### 1.3 Evaluation criteria

Postpartum hemorrhage volume was calculated using the volume method. Blood was collected with a postpartum blood collection container, and the hemorrhage volume was measured. Efficacy Evaluation Criteria: Effective: Vaginal bleeding volume ≤ 50 mL/h, bleeding significantly reduced or stopped, vital signs stable, urine output ≥ 30 mL/h. Ineffective: Vaginal bleeding volume > 50 mL/h, or uncontrollable bleeding, deteriorating vital signs, urine output < 30 mL/h.

### 1.4 Follow-up time and indicators

Follow-up was conducted via telephone or outpatient visits, with a follow-up duration ranging from 6 months to 5 years. Follow-up Indicators: Short-term follow-up (4 days and 42 days post-surgery), Color Doppler ultrasound and MRI were performed to assess uterine involution and ovarian conditions; 6-month and 1-year follow-up, Hysteroscopy was performed to assess the presence of intrauterine adhesions and endometrial damage; Recovery of menstruation, Follow-up to understand the recovery of ovarian function and uterine status; 2–5 years follow-up, Records included subsequent pregnancy status, pregnancy outcomes, mode of delivery, and the occurrence of postpartum hemorrhage (see Table 2).

**Table 2:** Follow-up time and indicators after uterine mesh compression suturing.

Indicators Follow-up time	US	MRI	Hysteroscopy	Menstrual recovery	Subsequent pregnancy and pregnancy outcome
4d	+	+			
42d	+	+			
6 m	+		+	+	
1Y				+	+
2Y					+
3Y					+
4Y					+
5Y					+

## 2 Results

After undergoing uterine mesh compression suturing, active uterine bleeding stopped within 10 min in all 45 patients with postpartum hemorrhage (PPH). No active vaginal bleeding was observed during a 30-min observation period, after which the abdomen was closed. The cumulative vaginal bleeding volume within 2 h postoperatively was <20 mL, indicating effective hemostasis (see Table 3). None of the patients showed signs of postoperative infection, and all abdominal incisions healed primarily (Grade A). Follow-up MRI performed 4 days after surgery showed scattered punctate mildly hyperintense signals in the myometrium, with no intrauterine fluid accumulation. MRI at 42 days postoperatively revealed no abnormal signals in the myometrium, a clearly visible endometrial line, and a uterus restored to normal size. Ultrasound at 42 days after surgery showed a uterus of normal size with homogeneous myometrial echogenicity and good involution. All 45 patients resumed normal menstruation 1–2 months after cessation of breastfeeding. Among them, 21 patients underwent hysteroscopy 6 months postoperatively, which showed no intrauterine adhesions, normal endometrial morphology, and clearly visible tubal ostia. During the 2–5 year follow-up period, 10 patients became pregnant again. Four of them delivered via repeat cesarean section without complications, with no placental abnormalities or postpartum hemorrhage occurring during subsequent deliveries. Six patients opted for induced abortion to terminate the pregnancy.

**Table 3:** Postoperative hemostatic effectiveness within 2 hours after uterine mesh compression suturing.

Observation time	Number of cases	Effective	Ineffective
10 min	45	+	-
30 min	45	+	-
2 h	45	+	-

## 3 Discussion

Postpartum hemorrhage (PPH) is a major threat to maternal life and remains the leading cause of maternal mortality worldwide. It continues to be a global clinical challenge. Current pharmacological and conservative surgical treatments aim, as far as possible, to preserve the uterus. The B-Lynch suture (6), introduced in the late 1990s, is a uterine compression technique used to manage PPH. Its application has markedly reduced PPH-related complications and the need for hysterectomy, thereby playing an important role in preserving maternal reproductive function. Since then, numerous modified techniques have been developed, including Cho suturing (7), Hayman suturing (8), Bhal suturing (9), Pereira suturing (10), Ouahba suturing (11), Hackethal suturing (12), and Mansoura-VV uterine compression suturing (13). These methods involve selective suturing at visible or suspected bleeding sites in the uterus; however, they are associated with several potential risks: (1) they may fail to achieve hemostasis at occult or late-onset bleeding sites; (2) localized compression may aggravate bleeding at other sites; and (3) the sutures may compress and cut through the myometrium, causing ischemia and potentially leading to uterine necrosis. In 2006, Treloar et al. (14) reported a case of uterine necrosis requiring hysterectomy 3 weeks after B-Lynch suturing. In addition, there have been reports from China (15) of uterine fistulas caused by infection secondary to inadequate uterine drainage after B-Lynch suturing. Gilberto (16) reviewed 104 cases of B-Lynch suturing in Brazil over a 15-year period and found that the main complications were related to blood transfusion and ICU admission; severe complications included puerperal infection, surgical site infection, and hysterectomy. The hysterectomy rate following B-Lynch suturing was 4.8%, with an effectiveness rate of 95.2% for controlling PPH.

The B-Lynch technique relies on a single suture that exerts a “band-like” compression on the uterine body, with the stress primarily distributed on the uterine surface and transmitted inward to compress the uterine cavity. In contrast, uterine mesh compression suturing uses 2–3 vertical mattress sutures passing through both the anterior and posterior uterine walls. Tightening these sutures creates a comprehensive, balanced, three-dimensional “mesh compression” system. This stress mesh encompasses the portion of the uterus affected by PPH and provides a continuous, uniform network of compressive forces to achieve mechanical hemostasis. The “mesh compression” acts only on the uterine wall while maintaining an empty uterine cavity, thereby avoiding impaired drainage due to external “bundling” of the uterus and reducing the risk of intrauterine adhesions and infection. The main characteristics of the “mesh compression” system are as follows: (1) compression is circumferential and balanced, and the direction of force is consistent with the centripetal involution of the uterus; (2) compression is confined to the uterine wall and does not restrict the uterine cavity, allowing physiological uterine emptying; and (3) the mesh sutures compress the arcuate vessels within the myometrium in a crisscross fashion rather than ligating the main uterine vessels. As the uterus involutes, the compressed uterine vessels can gradually re-open, thereby restoring normal uterine blood flow anatomy to the greatest extent possible.

When first introduced (17), uterine mesh compression suturing was primarily considered an alternative to hysterectomy. With accumulating experience, its indications have been progressively expanded. In patients with refractory PPH, we perform uterine mesh compression suturing and combine it with uterine artery ligation when necessary. According to current PPH guidelines (18), blood loss ≥1,500 mL is regarded as a threshold for second-line emergency interventions. In our current treatment strategy, PPH with blood loss ≥1,500 mL serves as the trigger for considering uterine mesh compression suturing. For patients whose blood loss exceeds 1,500 mL during cesarean section, or in whom B-Lynch suturing fails intraoperatively, we promptly perform uterine mesh compression suturing, with satisfactory hemostatic outcomes.

Among the 45 patients who underwent uterine mesh compression suturing in this study, no infections or hysterectomies occurred, and uterine preservation was achieved in all cases. Clinically, uterine bleeding ceased within 10 min after completion of the mesh suturing, indicating a rapid hemostatic effect. Earlier intervention and lower intraoperative blood loss were associated with reduced transfusion requirements and a lower likelihood of ICU admission, both intraoperatively and postoperatively. During the same procedure, uterine artery ligation or uterine cavity packing can be performed as adjunctive measures when indicated. Compared with B-Lynch suturing, uterine mesh compression suturing was associated with lower infection and hysterectomy rates. Follow-up showed satisfactory restoration of uterine morphology, and hysteroscopy confirmed a normal uterine cavity without adhesions. Long-term follow-up indicated good recovery of menstruation, and some patients subsequently conceived and delivered successfully.

An additional advantage of uterine mesh compression suturing is that it does not require detailed pre-assessment of suture feasibility. If manual uterine compression fails to reduce bleeding, mesh suturing can be initiated directly. In practice, placing two lines of mesh sutures typically requires approximately 8–10 min. For surgeons already familiar with the B-Lynch technique, uterine mesh compression suturing is relatively easy to master. Several technical points should be noted: (1) the first suture should cross the uterine fundus twice on the serosal surface, keeping an adequate distance from the uterine cornua to protect the tubal ostia; and (2) the suture should be tightened with sufficient but controlled tension to ensure effective compression while avoiding damage to the uterine serosa. After completion of the mesh suturing, the uterus assumes a characteristic “gyrus-like” appearance, which visually reflects the global “mesh compression” effect. This has been repeatedly confirmed in clinical practice. For patients with refractory PPH who do not respond to uterine massage, uterotonic agents, uterine cavity packing, uterine artery ligation, and B-Lynch suturing, uterine mesh compression suturing should be adopted decisively as an emergency measure. Alternatively, when intraoperative blood loss during cesarean section exceeds 1,500 mL, mesh compression suturing may be implemented directly. We believe that uterine mesh compression suturing represents a valuable alternative surgical option for treating refractory PPH and plays an important role in uterine preservation in this population.

This study has several limitations. First, it was a retrospective analysis with a relatively small sample size and a relatively short follow-up period. Therefore, the long-term efficacy and safety of uterine mesh compression suturing require further confirmation through extended follow-up and additional clinical data. Second, the study lacked randomized controlled trials and thus lacks high-level evidence-based support. In the future, with multicenter collaboration and a larger sample size, we plan to establish control groups to further clarify the indications for and surgical efficacy of uterine mesh compression suturing.

## Data Availability

The original contributions presented in the study are included in the article/Supplementary material, further inquiries can be directed to the corresponding author/s.
