# Supplementary figures and images for: Uterine mesh compression suturing for refractory postpartum hemorrhage: a retrospective study of 45 cases
Source: Front Med (Lausanne). 2025 Dec 12;12:1675709. doi: 10.3389/fmed.2025.1675709 (PMC12742309; doi:10.3389/fmed.2025.1675709)

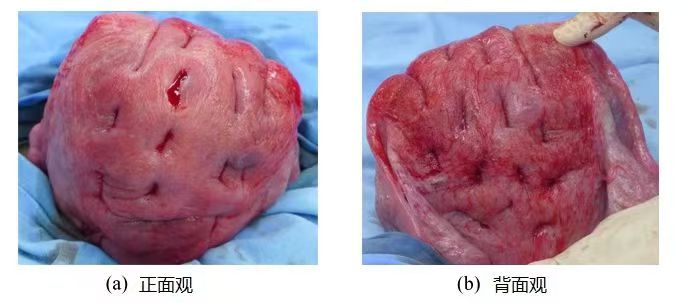

Supplement: Supplementary file 1 [file Image_1.jpeg]

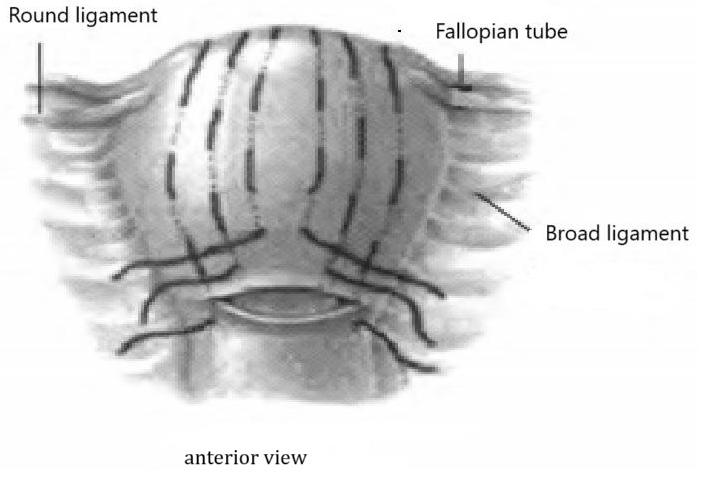

Supplement: Supplementary file 2 [file Image_2.jpeg]

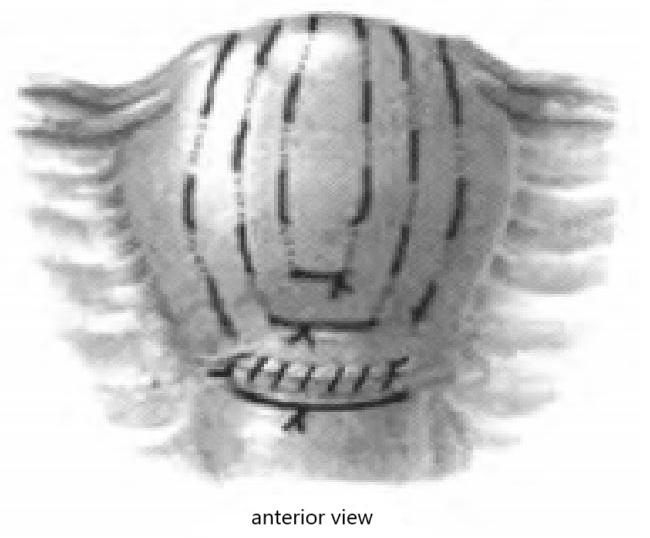

Supplement: Supplementary file 3 [file Image_3.jpeg]

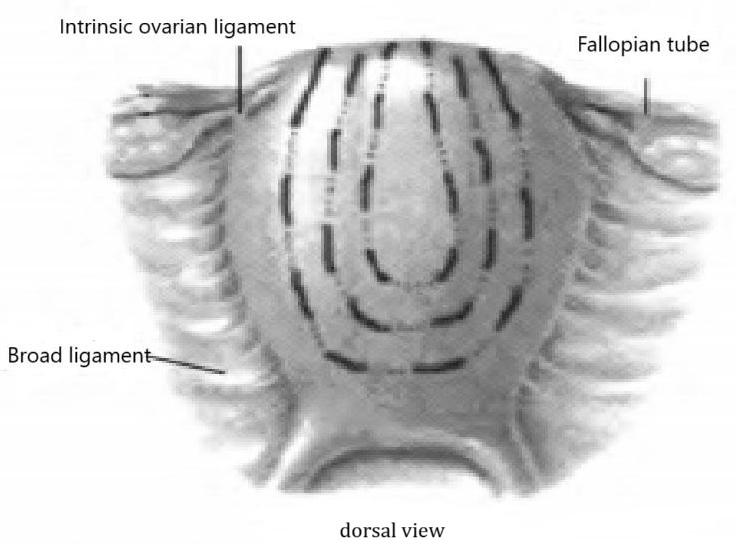

Supplement: Supplementary file 4 [file Image_4.jpeg]

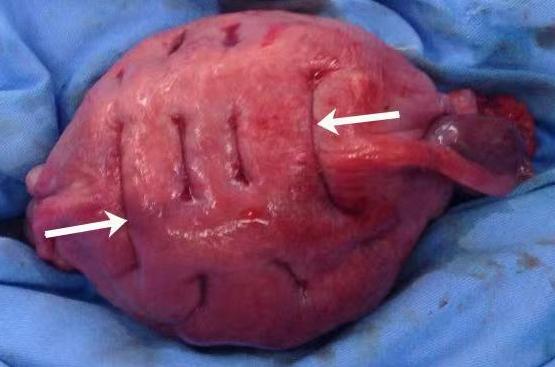

Supplement: Supplementary file 5 [file Image_5.jpeg]
